# Anthracycline Shunt Metabolites From Philippine Marine Sediment-Derived *Streptomyces* Destroy Cell Membrane Integrity of Multidrug-Resistant *Staphylococcus aureus*

**DOI:** 10.3389/fmicb.2020.00743

**Published:** 2020-04-24

**Authors:** Melissa June V. Paderog, Angelica Faith L. Suarez, Edna M. Sabido, Zhen Jie Low, Jonel P. Saludes, Doralyn S. Dalisay

**Affiliations:** ^1^Department of Pharmacy, College of Health and Allied Medical Professions, University of San Agustin, Iloilo City, Philippines; ^2^Center for Chemical Biology and Biotechnology (C2B2), University of San Agustin, Iloilo City, Philippines; ^3^Center for Natural Drug Discovery and Development (CND3), University of San Agustin, Iloilo City, Philippines; ^4^Waters Pacific Pte Ltd, Singapore, Singapore; ^5^Department of Chemistry, College of Liberal Arts, Sciences, and Education, University of San Agustin, Iloilo City, Philippines; ^6^Balik Scientist Program, Philippine Council for Health Research and Development, Department of Science and Technology, Taguig, Philippines; ^7^Department of Biology, College of Liberal Arts, Sciences, and Education, University of San Agustin, Iloilo City, Philippines

**Keywords:** antibacterial, Philippine marine sediments, anthracyclines shunt metabolites, multidrug-resistant *Staphylococcus aureus* (MDRSA), *Streptomyces*, cell membrane integrity, Bisanhydroaklavinone, 1-hydroxybisanhydroaklavinone

## Abstract

The rise of antibiotic resistance (ABR) and the drying up of the pipeline for the development of new antibiotics demands an urgent search for new antibiotic leads. While the majority of clinically available antibiotics were discovered from terrestrial *Streptomyces*, related species from marine sediments as a source of antibiotics remain underexplored. Here, we utilized culture-dependent isolation of thirty-five marine sediment-derived actinobacterial isolates followed by a screening of their antibacterial activity against multidrug-resistant *S. aureus* ATCC BAA-44. Our results revealed that the crude extract of *Streptomyces griseorubens* strain DSD069 isolated from marine sediments collected in Romblon, Philippines displays the highest antibacterial activity, with 96.4% growth inhibition. The *S. aureus* ATCC BAA-44 cells treated with crude extract of *Streptomyces griseorubens* strain DSD069 showed cell membrane damage as demonstrated by (a) leakage and loss of vital cell constituents, including DNA and proteins, (b) irregular shrinkage of cells, and (c) increase membrane permeability. The antibiotic compounds were identified as Bisanhydroaklavinone and 1-Hydroxybisanhydroaklavinone with MIC value of 6.25 μg/mL and 50.00 μg/mL, respectively. Bisanhydroaklavinone and 1-Hydroxybisanhydroaklavinone are shunt metabolites in the biosynthesis of anticancer anthracycline derivatives namely doxorubicin, daunorubicin, and cinerubins. It is rare, however, that shunt metabolites are accumulated during fermentation of marine sediment-derived *Streptomyces* strain without genetic modification. Thus, our study provides evidence that natural bacterial strain can produce Bisanhydroaklavinone and 1-Hydroxybisanhydroaklavinone as antibiotic leads to combat ABR.

## Introduction

With the increasing emergence of antibacterial resistance (ABR) among common pathogens, the quest to discover novel and new antibiotics is, therefore, urgent. ABR is the natural ability of bacteria to resist the effects of antibiotics, thereby rendering antibiotics less effective (Khan and Khan, 2016; Vestergaard et al., 2019). Various resistance mechanisms such as enzyme mutations, multidrug efflux-mediated resistance, production of antibiotic degrading enzymes, and modification of drug binding sites were developed by bacteria as their defense against antibiotics (Sriramulu, 2013; Vestergaard et al., 2019). These resistance mechanisms are developed rapidly, and the spread of multidrug-resistant strains is uncontrollable. Unfortunately, the ABR crisis is coupled with shortage of new antibiotics to replace those rendered ineffective by ABR strains. Therefore, urgent efforts to discover new antibiotics from underexplored sources are needed.

Members of the genus *Streptomyces* of the phylum Actinobacteria are soil saprophytes that are known producers of antibiotics (Shaik et al., 2017; Yang and Song, 2017; Kemung et al., 2018; Yang et al., 2020). These microorganisms contain high GC content in their DNA sequences and are reported to have antibiotic-producing biosynthetic gene clusters (BGC) (Hopwood, 2007; Dhakal et al., 2017; Romano et al., 2018) that produces about 75% of the clinically available antibacterial drugs in the market (Kemung et al., 2018). However, in the last 20 years, the re-discovery of previously characterized bioactive compounds and strain redundancy decreased the interest in these soil-dwelling bacteria as a source of novel bioactive compounds (Yang and Song, 2017; Almeida et al., 2019). Thus, *Streptomyces* living in other niches, such as the marine environment, gained value because of their chemodiversity (Chelvan et al., 2016; Dhakal et al., 2017; Yang and Song, 2017; Kemung et al., 2018; Romano et al., 2018; Al-Dhabi et al., 2019; Almeida et al., 2019).

In response to the ABR crisis and the challenges of finding new antibiotics, we collected marine sediments near Romblon, Philippines as a source of marine-derived Actinobacteria. We focused on isolating the Actinobacteria from marine sediments and screen their crude extracts against *Staphylococcus aureus* ATCC BAA-44, profiling antibacterial activity and determining their membrane disruption ability, and identifying the compound(s) responsible for antibiotic activity. We performed a culture-dependent isolation approach using the dry stamp and heat shock methods (Mincer et al., 2002; Jensen et al., 2005; Dalisay et al., 2013) utilizing minimal marine media to isolate marine sediment-derived Actinobacteria. We isolated 35 actinobacterial isolates with six isolates (17% hit rate) active against *S. aureus* ATCC BAA-44. *Streptomyces griseorubens* strain DSD069 showed the highest antibiotic activity. Its crude extract caused cell membrane damage and intracellular leakage, leading to compromised cell membrane integrity and death of *S. aureus* ATCC BAA-44. The antibiotic compounds were accumulated in the growth medium during fermentation, which were later identified as shunt metabolites in the biosynthesis of anticancer anthracycline derivatives such as doxorubicin, daunorubicin, and cinerubins. This work is the first report on the accumulation of anticancer anthracycline derivatives shunt metabolites by Philippine marine sediment-derived *Streptomyces* strain without genetic modification. Thus, our study provides evidence that natural bacterial strain can produce Bisanhydroaklavinone and 1-Hydroxybisanhydroaklavinone as antibiotic leads to combat ABR.

## Materials and Methods

### Sample Collection and Culture-Dependent Isolation of Marine Actinobacteria

Sediment samples were collected from six sites near Alad and Lugbong islands of Romblon, Philippines. The sediments were collected 200–500 m away from the islands by SCUBA at a depth of 20–30 m. The sediment samples were placed in sterile cylindrical tubes and kept at 4°C until further processing. Dry stamp method (DSM) and heat shock method (HSM) (Mincer et al., 2002; Jensen et al., 2005; Dalisay et al., 2013) using selective minimal marine media (ISP4 and noble agar) (Dalisay et al., 2013) were used to grow the marine sediment-derived Actinobacteria. The inoculated plates were incubated at room temperature for 30 to 60 days.

### Morphological Characterization of Marine Sediment-Derived Actinobacteria

Marine sediment-derived Actinobacteria were examined morphologically in terms of their mycelium production, specifically substratum mycelium (pigmentation) and aerial mycelium (spores). Spore size was measured using scanning electron microscopy (SEM). The spores were washed twice with PBS (0.1 M, pH 7.4), centrifuged, and fixed with glutaraldehyde solution (2.5% in PBS) for 1.5 h at 4°C. The fixed spores were dehydrated using gradient concentration of ethanol (30, 50, 85, 95, 100%) and twice with *t*-butyl alcohol before freezing at −20°C for 5 min. The spores were viewed under a scanning electron microscope, SEM (JEOL JSM 5510LV) (Fatima et al., 2019).

### Extraction of Secondary Metabolites

Actinobacterial isolates were grown in marine medium 1 (MM1) agar (Dalisay et al., 2013) plates. Successive re-plating was done to obtain a pure isolate. Large-scale fermentation of the isolates was performed according to the method of Dalisay et al. (2013). Secondary metabolites were extracted using EtOAc and the extract partitioned with water. The EtOAc fractions were concentrated *in vacuo* and subsequently stored at −80°C until further use.

### Multidrug-Resistant *Staphylococcus aureus* ATCC BAA-44

*Staphylococcus aureus* ATCC BAA-44 is a particular strain containing the SCC*mec* Type I gene. It is resistant to 18 clinically significant antibiotics namely ampicillin, amoxicillin/clavulanic acid, ciprofloxacin, cephalothin, doxycycline, gentamicin, erythromycin, imipenem, methicillin, penicillin, tetracycline, oxacillin, azithromycin, clindamycin, ceftriaxone, rifampin, amikacin, and tobramycin [Staphylococcus aureus subsp. aureus Rosenbach (ATCC^®^ BAA-44^TM^), 2019].

### Antibacterial Activity Assay

#### Agar Well Diffusion Assay

The preliminary screening of extracts against *S. aureus* ATCC BAA-44 was performed using agar well diffusion assay. The *S. aureus* ATCC BAA-44 (1 × 10^6^ CFU/mL) in agar medium was prepared by pour plate technique. Thereafter, three wells were punched on the agar. The first well was filled with tetracycline (0.050 mg/well) as a positive control. The second well was filled with DMSO as a negative control, and the third well was filled with Actinobacteria crude extracts (2.0 mg/well). The samples were allowed to diffuse unto the agar for 1 h before incubation at 37°C for 18–24 h. Zone of inhibition was measured in mm after the incubation period. Extracts that exhibited zone of inhibition of more than 4.0 mm were considered to have antibacterial activity (Bredholt et al., 2008; Valli et al., 2012).

#### Microbroth Susceptibility Assay

The *S. aureus* ATCC BAA-44 (1 × 10^6^ CFU/mL) in Mueller-Hinton broth (MHB) was prepared in a 96-well plate and exposed to different treatments [tetracycline 0.25 mg/mL in DMSO (positive control), DMSO (negative control), and crude extracts (2.5 mg/mL of DMSO)] for 18–24 h at 37°C. The bacterial density at OD 620 nm was measured after incubation using a microplate reader (Multiskan^TM^ FC Thermo Fisher) and percent growth inhibitions were calculated.

Extracts with more than 50% growth inhibition were confirmed for antibacterial activity. The extract with the highest % growth inhibition against the multidrug-resistant *S. aureus* ATCC BAA-44 was further subjected to antibacterial activity profiling, mechanism of action assay, and chemical profiling for antibiotic compound identification (Langfield et al., 2004; Delgado-Andrade et al., 2006; Dalisay et al., 2013; Hayashi et al., 2014).

### 16S rRNA Gene Amplification and Sequencing

Actinobacterial DNA was extracted using the DNeasy Blood and Tissue Kit (Qiagen) according to the manufacturer’s instruction. For 16S rRNA Gene amplification, universal primers 27F (5′-AGAGTTTGATCCTGGCTCAG-3′) and 1492R (5′-TACGGCTACCTTGTTACGACTT′) were used in PCR reaction. The reaction mixture (50 μL) contained 5 μL Taq Polymerase (5 units/μL), 5 μL MgCl_2_ (50 mM), 5 μL Taq Buffer, 5 μL dNTPs (10 mM), 3 μL 27F primer (10 mM), 3 μL 1492R primer (10 mM), 19 μL nuclease-free H_2_O (ultrapure water) and 100 ng/μL DNA template. The PCR conditions were as follows: initial denaturation at 98°C for 3 min; 35 cycles at 98°C for 10 s, 60°C for 10 s and 72°C for 60 s; and a 10-min final extension at 72°C (T100^TM^ BioRad Thermal Cycler). The amplified products were cleaned using the QIAquick PCR cleanup kit (Qiagen) according to the manufacturer’s protocol. The universal primers 27F, 518F, 800R, and 1492R were used to obtain the 1300–1500 bp gene sequences. The species-level affiliation of the sequenced 16S rRNA gene was determined using BLAST (Altschul et al., 1990). The phylogenetic tree was constructed using multiple alignments with maximum likelihood method in Mega 7.0 software (Kumar et al., 2016). Bootstrap analysis based on 1,000 replicates evaluated the resulting tree topologies.

### Antibacterial Activity Profiling

#### Time-Kill Kinetics Assay

Following the same procedure in microbroth susceptibility, the assay was performed using 0.25 mg/mL tetracycline (positive control), DMSO (negative control), and 2.5 mg/mL of most active extract as treatments. In this assay, measurement of bacterial densities was obtained every 6 h (0, 6, 12, 18, and 24 h) within a 24 h period using microplate reader (Multiskan^TM^ FC Thermo Fisher). Percent bacterial growth inhibition of the treatments at different time points was calculated and plotted (Tenover et al., 2004; Peck et al., 2012).

#### Minimum Inhibitory Concentration (MIC)

Two-fold serially diluted concentrations of extract (dilution range of 625 – 0.61 μg/mL) and tetracycline (dilution range of 62.5 – 0.061 μg/mL) was utilized, with DMSO as the negative control. The test bacterial strain (1 × 10^6^ CFU/mL) was exposed to different treatments in a 96-well plate for 18–24 h at 37°C. The bacterial densities of different concentrations were measured at OD 620 nm using microplate reader (Multiskan^TM^ FC Thermo Fisher). The corresponding % growth inhibition was calculated to identify the minimum concentration that could inhibit 90% of bacterial growth (du Toit and Rautenbach, 2000; Andrews, 2001; Delgado-Andrade et al., 2006).

Although tetracycline was listed as one of the antibiotics with resistance against *S. aureus* ATCC BAA-44, this antibiotic was used as the positive control in all the antibacterial assays covered in this work. We found that *S. aureus* ATCC BAA-44 has an MIC value of 31.25–50 μg/mL for tetracycline according to the protocol described in this work. Tetracycline susceptibility to *S. aureus* ATCC BAA-44 was also observed by other groups where MIC values were reported at 16 μg/mL (Rogers et al., 2010) and 64 μg/mL (Podoll et al., 2013).

#### Membrane Leakage Assay

Overnight grown broth culture of *S. aureus* ATCC BAA-44 (0.2 OD 620 nm) was prepared. Subsequently, the bacterial inoculum was exposed for 18–24 h to different treatments, namely 5% Tween 80 (positive control), DMSO (negative control), and the most active extract with concentrations of 2.50, 1.25, and 0.625 mg/mL in DMSO. The measurement of proteins and DNA that leaked out of the cell membrane was measured using a UV-Vis spectrophotometer (Shimadzu UV1280 Spectrophotometer) (Thombre et al., 2016; Tiwari et al., 2018). Leaked DNA was measured at 260 nm according to the method of Zhang et al. (2017), and proteins were measured by modifying the procedures of Bradford (1976). Here, proteins were directly measured at 280 nm instead of colorimetric measurement.

#### Transmission Electron Microscopy (TEM)

Bacterial cells of *S. aureus* ATCC BAA-44 (0.6 OD 620 nm) were exposed to the extract at 2.5 mg/mL concentration and DMSO (negative control) for 24 h incubation period. Untreated bacterial cells were also prepared as a control. The extract-treated, DMSO-treated, and untreated cells were then processed to adhere to an aluminum film and then washed thrice with phosphate buffer saline (PBS) with a concentration of 0.1 M (pH 7.4). After washing, the cells were stained with 2% phosphotungstic acid and subjected to dehydration with a gradient concentration of ethanol (30, 50, 85, 95, 100%). The dehydrated cells were washed twice with *t*-butyl alcohol before freezing it at −20°C for 5 min. The cells were then placed in the TEM sample holder and coated with gold using a sputter. The cells were viewed using TEM (JEOL JEM 1010) (Schmid and Sakamoto, 2001; Karsha and Lakshmi, 2010; Bai et al., 2015).

#### Flow Cytometry

Overnight broth culture of *S. aureus* ATCC BAA-44 was utilized. The cells were harvested by centrifugation (4500 rpm for 5 min) and re-suspended in PBS (7.4 pH) to a final bacterial density of 0.05 OD 600 nm. The bacterial suspension in PBS was transferred to a 96-well plate. The bacterial suspensions were treated with vancomycin (7.5 mg/mL), DMSO, and active extract (2.5 mg/mL). Untreated cells (only bacterial suspension, negative control) and 70% ethanol-treated cells as positive control to represent dead cells were transferred onto their corresponding wells in 96-well plate. The plate was incubated for 4 h at 37°C. After incubation, the cells were centrifuged at 4500 rpm for 5 min and re-suspended in dye solution (optimized concentrations of the Live/Dead Cell Double Staining Kit of Sigma-Aldrich). Single stained cells were also prepared for fluorescence compensation in the flow cytometer. The plate was incubated at 37°C for 15 min (Nowakowska et al., 2015). After the incubation period, the cells were centrifuged (4500 rpm for 5 min) and resuspended in 100 μL PBS. Data were then acquired using Amnis^TM^ FlowSight (Merck KGaA) imaging flow cytometer equipped with a 488 nm laser. Six thousand (6000) cell events were acquired per replicate, and 87% cell events were analyzed to eliminate cell debris. Propidium iodide fluorescence was measured at 642–745 nm band (Channel 5), and calcein fluorescence was measured at 505–560 nm band (Channel 2). The analysis was done using IDEA.6.2.188. Release.86x application software considering zero rfu as the lowest fluorescence unit. Propidium iodide fluorescing cells (R1) were gated as dead cells with permeant cell membranes, and calcein fluorescing cells (R2) were gated as live cells (Wu et al., 2016). The experiment was performed in triplicates.

### Chemical Profiling

#### Thin-Layer Chromatography Profiling

Thin-layer chromatography (TLC) profile of the most active extract was carried out using pre-coated TLC plates (Merck TLC Silica Gel 60 W F254s–normal phase) and methanol–dichloromethane (1:13) as solvent system through one-way ascending technique. The developed chromatogram was visualized under UV light at 365 and 254 nm and retention factor (R_f_) values of each band were calculated (Jayashree et al., 2014). Direct bioautography of the chromatogram was further performed by covering the surface of the TLC plate with a soft agar (MHA 0.8% agar) seeded with 1 × 10^6^ CFU/mL of *S. aureus* ATCC BAA-44 and incubated overnight. The zone of inhibition of antibacterial components was visualized by detecting mitochondrial reductase enzyme activity of viable cells using a resazurin reduction-based reaction. The metabolically active viable cells convert resazurin (blue colored compound) to its reduced form resorufin (pink colored compound). Thus, the zone of inhibition appears as blue spot over a pink background (Choma and Grzelak, 2010; Choma and Jesionek, 2015; Jesionek et al., 2015).

#### Chromatographic Purification of Bioactive Compound

Guided by UV activity of bioactive bands in TLC-bioautography, the crude extract of the most active actinobacterial isolate was purified through a silica column (0.5 mm diameter × 54 mm height). Dichloromethane (DCM) followed by methanol-dichloromethane (1:13) was used as the mobile phase. Collected fractions were spotted onto a silica TLC plate and developed using methanol-dichloromethane (1:13). The fractions containing bioactive bands based on UV activity were pooled together and dried using a vacuum concentrator (Eppendorf^TM^ Vacufuge^TM^ Concentrator) at V-HV mode, 30°C for 2 h). Subsequently, the dried bioactive fraction was developed on a silica TLC plate using methanol-dichloromethane (1:13) as solvent. The identified bioactive bands based on R_f_ values in the TLC profile were isolated by preparative-TLC method using methanol-dichloromethane (1:13) solvent system.

#### LCMS Analyses of Bioactive Fraction

Mass spectrum for dereplication analysis were acquired using high-resolution mass spectrometer (Waters Xevo^®^ G2-XS QTOF) equipped with high-performance system, StepWave^TM^ ion source technology, XS collision cell technology, QuanTOF^TM^ technology, UPLC/TOFMR for high transmission mode, and UPLC/MS^e^ for data acquisition. The solvent system was water with 0.1% (v/v) formic acid (solvent A) and acetonitrile with 0.1% (v/v) formic acid (solvent B). The analysis was performed with flow rate of 0.5 mL/min with an oven temperature at 40°C. The interface used for MS^e^ was electron spray ionization (ESI) with a collision energy of 6.0 eV for low energy and 10.0 eV for high energy. The scan time was set to 0.250 s using polarity positive mode with a mass range scan of m/z 50–900, and using a single UV wavelength of 254 nm. MS data were analyzed using Waters UNIFI Scientific Information System^®^ and Chemspider^TM^ Database.

## Results and Discussion

Members of the phylum Actinobacteria, which is exemplified by the genus *Streptomyces*, are well-known to produce bioactive compounds with various biological and pharmaceutical importance. Most of these compounds were isolated from terrestrial sources (Barka et al., 2016; Van Der Meij et al., 2017). However, there were reports recently, where their marine counterparts were also recognized as a source of bioactive compounds (Shaik et al., 2017; Yang and Song, 2017; El-Gendy et al., 2018; Kemung et al., 2018; Subramani and Sipkema, 2019). The condition in the marine environment is remarkably different from that of the terrestrial environment. It is a hostile niche (Aneiros and Garateix, 2004; Imada, 2005; Valli et al., 2012), where marine microorganisms are exposed to low nutrition, high salinity, and high-pressure (Soliev et al., 2011; Almeida et al., 2019). As a result, marine microorganisms developed unique metabolic and physiological ability to survive by activating their environment adaptation genes (ENAs) (Almeida et al., 2019). Because of this, they produce different secondary metabolites that their terrestrial counterparts do not have (Imada, 2005; Zheng et al., 2011; Dholakiya et al., 2017).

In the last decade, marine sediments have become reference hotspots for bioprospecting of marine *Streptomyces* with antibiotic activities (Kamjam et al., 2017; Yang et al., 2020). Recent reports described that marine sediment-derived *Streptomyces* species are producers of diverse antibiotic compounds. This includes 2-alkyl-4-hydroxyquinoline antibiotics and antifungals produced by *Streptomyces* sp. strain MBTG13 isolated from sediments of Jeju Island, South Korea (Kim et al., 2019), polycyclic polyether compounds, terrosamycins A and B, produced by *Streptomyces* sp. RKND004 isolated from sediments of Prince Edward Island, Canada (Sproule et al., 2019), and oxazole-triene compound, inthomycin B, produced by *Streptomyces* sp. YB104 isolated from deep sediments in South Atlantic Ocean (Wu et al., 2018). These reports imply that *Streptomyces* thriving in underexplored niches such as marine sediments are resource for potential antibacterial compounds that are of diverse chemical characteristics.

As part of our natural drug discovery program for new antibiotics, we recovered 35 actinobacterial isolates from marine sediment samples collected near Romblon islands, Philippines using a culture-dependent isolation technique in selective minimal marine media (Mincer et al., 2002; Jensen et al., 2005; Dalisay et al., 2013). In this approach, we utilized two media (noble agar and ISP4) with components that mimic the sea water nutrients, pH, and salinity. Further, the media contain cyclohexamide to inhibit fungal contamination and the antibiotic rifamycin to select Actinobacteria (Mincer et al., 2002; Jensen et al., 2005; Hames-Kocabas and Uzel, 2012; Dalisay et al., 2013; Claverias et al., 2015; Jiang et al., 2016; Jose and Jha, 2017; Subramani and Sipkema, 2019).

The morphological characteristics of the actinobacterial isolates were profiled according to their aerial (spores) and substratum (pigmentation) mycelium (Table 1). Results showed diverse substratum mycelium production ranging from no pigmentation to yellowish, red-orange to red, and light yellowish-orange to brown. Although recovered from the same collection site, the isolates have varied growth period. Some (4 out of 35 isolates, 11%) grew for as fast as 33 days, while 14% (5 out of 35 isolates) were slow growers that required more than 100 days to develop. In terms of culture-dependent isolation, 29 isolates (82.9%) were recovered using dry stamp method while only six isolates (17.1%) were recovered using heat shock method. Besides, 60% (21 isolates) grew well in the noble agar medium while only 40% (14 isolates) grew well in the ISP4 medium. Our results revealed that the isolates in this study are diverse, as shown by the various diffusible pigments they produced, as well as growth variation in minimal marine media.

**TABLE 1:** Isolation profile of marine sediment-derived actinobacterial strains.

**Code**	**Collection site**	**Method of isolation**	**Incubation Period (days)**	**Medium for isolation and description of colonies**		
				**Medium**	**Mycelium**	
					**Aerial**	**Substratum**
DSD044	Lugbung	DSM	33	Noble agar	Grayish-white	Red-orange
DSD045	Lugbung	DSM	33	Noble agar	Gray	Red-orange
DSD046	Lugbung	DSM	33	Noble agar	Gray	Red-orange
DSD047	Lugbung	DSM	33	Noble agar	Gray	None
DSD048	Lugbung	HSM	68	Noble agar	Gray	None
DSD049	Lugbung	HSM	68	Noble agar	Grayish-white	Red
DSD050	Lugbung	HSM	68	Noble agar	Gray	Light yellow-brown
DSD051	Lugbung	HSM	68	Noble agar	Gray	Light yellow-brown
DSD052	Lugbung	HSM	68	Noble agar	Gray	Light yellow-brown
DSD053	Lugbung	HSM	68	Noble agar	Gray	Red
DSD054	Alad	DSM	42	ISP4	Gray with some white portion	Brown
DSD055	Lugbung	DSM	42	ISP4	Gray	Yellowish-brown
DSD056	Lugbung	DSM	34	ISP4	Grayish with white portion	Yellowish-brown
DSD057	Lugbung	DSM	34	ISP4	Grayish with white portion	Yellowish-brown
DSD058	Lugbung	DSM	43	ISP4	Grayish with white portion	Yellowish
DSD059	Lugbung	DSM	43	ISP4	Grayish with white portion	Yellowish-brown
DSD060	Lugbung	DSM	43	ISP4	Gray	Yellowish
DSD061	Lugbung	DSM	43	ISP4	Gray	Yellowish
DSD062	Lugbung	DSM	43	ISP4	Gray	Yellowish
DSD063	Lugbung	DSM	43	ISP4	Gray	Yellowish
DSD064	Alad	DSM	34	Noble agar	Grayish-white	Yellowish-brown
DSD065	Alad	DSM	34	Noble agar	Gray	Yellowish-brown
DSD066	Alad	DSM	34	Noble agar	White	Yellowish
DSD067	Alad	DSM	34	Noble agar	White	Yellowish-brown
DSD068	Alad	DSM	34	Noble agar	Gray	None
DSD069	Lugbung	DSM	47	ISP4	Gray	None
DSD070	Alad	DSM	59	ISP4	Gray	Red
DSD071	Alad	DSM	103	Noble agar	White	Brownish-yellow
DSD072	Alad	DSM	103	Noble agar	White	Brownish
DSD073	Alad	DSM	103	Noble agar	White	Brownish
DSD074	Alad	DSM	67	ISP4	Gray	Red
DSD075	Alad	DSM	67	ISP4	Gray	Red
DSD076	Alad	DSM	103	Noble agar	White	Brownish
DSD077	Alad	DSM	103	Noble agar	White	Brownish
DSD078	Alad	DSM	67	Noble agar	Grayish white	Red

*DSM, dry stamp method; HSM, heat shock method.*

### Antibacterial Activity Screening of Crude Extracts

The crude extracts of actinobacterial isolates (Supplementary Figure 1) were initially screened for antibacterial activity through agar well diffusion assay against *S. aureus* ATCC BAA-44. The results demonstrated that out of 35 crude extracts, seven (20%) showed antibacterial activity against *S. aureus* ATCC BAA-44 that exhibited zones of inhibition ranging from 4.1–15.0 mm (Table 2). Isolate DSD069 exhibited the highest zone of inhibition of 15.0 mm (±0.2 SD) at 2.0 mg/well, which is nearly equal to the activity of tetracycline at 14.0 mm (±0.1 SD) at 0.05 mg/well.

**TABLE 2:** Agar well diffusion assay against multidrug-resistant *S. aureus*

**Treatments**	**Zone of Inhibition (mm)**
Tetracycline	14.0 ± 0.1
DSD047	4.1 ± 0.2
DSD061	8.0 ± 2.0
DSD062	5.9 ± 0.1
DSD065	5.8 ± 0.7
DSD067	4.3 ± 0.5
DSD069	15.0 ± 0.2
DSD070	4.8 ± 0.4

*Data presented are mean zone of inhibition (mm) ± SD (standard deviation) of the bioactive marine sediment-derived actinobacterial isolates against *S. aureus* ATCC BAA-44 (1 × 10^6^ CFU/mL) through agar well diffusion assay. Cultures of the test bacterial strains in Mueller-Hinton agar plates were exposed to treatments namely, *Actinobacterial* crude extract (2 mg/well), tetracycline - positive control (0.05 mg/well) and DMSO (negative control) for 18–24 h. The experiment was performed in three trials.*

**FIGURE 1:**
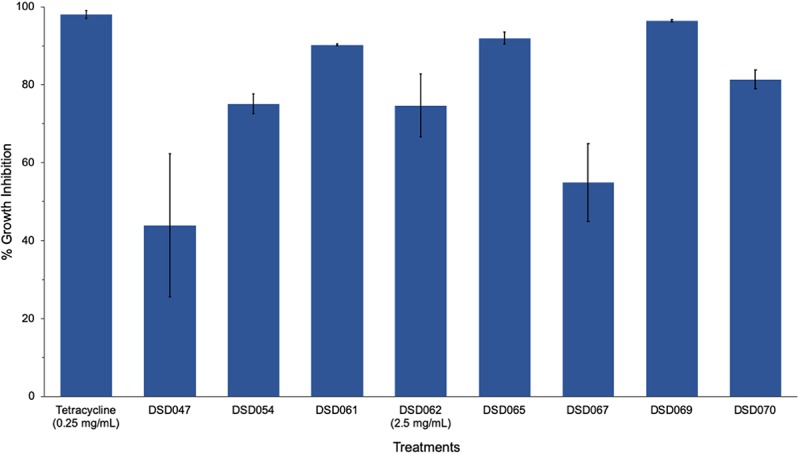
Antibacterial activity of bioactive actinobacterial isolates against multidrug-resistant *Staphylococcus aureus*. Data illustrated are the percent growth inhibition (± SD) of the antibacterial isolates against *S. aureus* ATCC BAA-44 evaluated through microbroth susceptibility assay. Cultures of test bacterial strain (1 × 10^6^ CFU/mL) were exposed to different actinobacterial extracts (2.5 mg/mL), positive control tetracycline (0.25 mg/mL), and DMSO (negative control) for 24 h. The percent growth inhibition were determined at 620 nm using the microtiter plate reader. The experiment was done in triplicates and performed in three trials.

The results obtained from well-diffusion assay were further validated by microbroth susceptibility assay. Out of the seven crude extracts found active in well-diffusion assay, six extracts showed more than 50% growth inhibition (Figure 1) in microbroth susceptibility assay as confirmatory screening. Isolate DSD069 crude extract was found to show higher growth inhibition of 96.40% at 2.5 mg/mL against *S. aureus* ATCC BAA-44 in comparison to tetracycline that showed 95.48% at 0.25 mg/mL. Isolate DSD069 was selected for subsequent tests not only for its confirmatory high antibacterial activity against *S. aureus* ATCC BAA-44 but also for its broad antibacterial activities against eight target bacteria (Supplementary Table 1). Investigation of the antibacterial compounds against these target bacteria is ongoing and will be reported in due course.

### 16S rRNA Gene Sequence of Isolate DSD069

**FIGURE 2:**
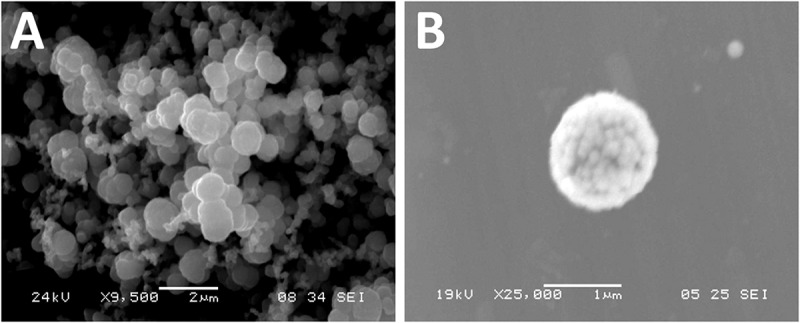
Scanning electron photomicrograph of marine sediment-derived *Streptomyces griseorubens* strain DSD069. **(A)** Photomicrograph of spores at 9,500x magnification, and **(B)** photomicrograph of spore at 25,000x magnification.

The identity of isolate DSD069 was determined using its 16S rRNA gene sequence. Using BLAST, the nearly complete 16S rRNA gene sequence (1411 bp) revealed 99.93% gene homology with *Streptomyces griseorubens* strain NBRC 12780, which was corroborated with the results obtained in phylogenetic analysis using Molecular Evolutionary Genetics Analysis (MEGA, V7) (Supplementary Figure 2). This result indicates that isolate DSD069 is a marine-derived *Streptomyces griseorubens* strain. The nearly complete 16S rRNA gene sequence was deposited in the GenBank with accession number MN818600. *S. griseorubens* strain DSD069 produced gray spores and no diffusible pigment when grown in marine ISP4 medium. SEM analysis of the spores revealed globular shape with size ranging from 1 to 2 μm (Figure 2).

*Streptomyces griseorubens* is a soil-derived actinobacterium (Pridham et al., 1957) that is involved in carbon and nitrogen recycling (Feng et al., 2014). Further, *S. griseorubens* possesses antibacterial activity against *Pseudomonas aeruginosa, S. aureus, Escherichia coli, S. pneumoniae*, and *Bacillus subtilis* (Al-Askar et al., 2014). A recent report described two lipocyclopeptide antibiotic compounds produced via non-ribosomal polypeptide synthetase (NRPS) biosynthetic gene cluster (BGC) in *Streptomyces griseorubens* strain INA 00887 (Tyurin et al., 2018). These antibiotics, namely, cryst-1 (Aspartocin B and C) and cryst-2 (Aspartocin A), are two major components of crystallomycin complex, an antibiotic discovered 60 years ago when advanced analytical tools were not available to fully characterize the compounds (Tyurin et al., 2018). Although *S. griseorubens* is known to be terrestrial, a *S. griseorubens* strain was previously isolated from marine sediments collected from the seabed in Weihai, China (Ye et al., 2009). Interestingly, the strain required seawater for growth and biosynthesis of anticancer metabolites, indicating that the strain has evolved and developed genes for marine adaptation (i.e., marine adaptation genes, MAG) as a mechanism to adapt in marine environment (Ye et al., 2009). The same adaptive mechanism may possibly have contributed to the survival of *S. griseorubens* strain DSD069 in the marine environment, thus allowing to produce antibiotic metabolites.

### Antibacterial Activity Profile of *Streptomyces griseorubens* Strain DSD069

**FIGURE 3:**
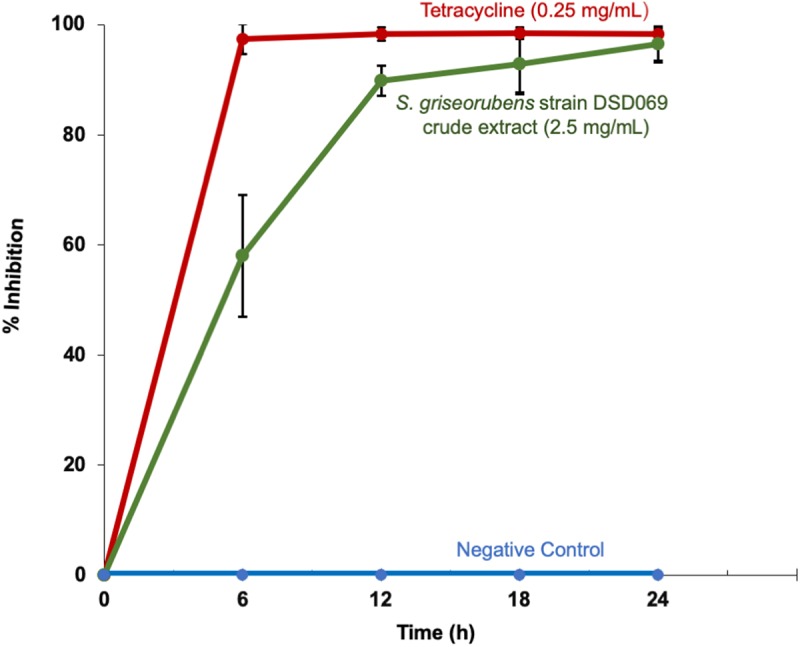
Time-kill kinetics against multidrug-resistant *S. aureus* ATCC BAA-44. Data presented are the % growth inhibition (± SD) of the treatments at different time points. *S. aureus* ATCC BAA-44 (1 × 10^6^ CFU/mL) was exposed to *S. griseorubens* strain DSD069 (2.5 mg/mL), 0.25 mg/mL tetracycline (positive control), and DMSO (negative control) for 24 h. Bacterial cell density was measured and corresponding % growth inhibition was calculated every 6 h interval from time 0 until 24 h incubation. The experiment was done in triplicates and performed in three trials.

Our results in the time-kill kinetics assay of the *S. griseorubens* strain DSD069 crude extract at 2.5 mg/mL against *S. aureus* ATCC BAA-44 showed a time-dependent antibacterial action, with a similar killing trend with that of tetracycline (0.25 mg/mL, positive control) (Figure 3). The crude extract (2.5 mg/mL) exhibited minimal antibacterial activity (58.11%) after 6 h incubation time then gradually increased (89.59%) after 12 h and reached maximum inhibition (96.44%) after 24 h incubation period.

When tested by microbroth dilution assay to determine its minimum inhibitory concentration (MIC), the crude extract showed 2.44 μg/mL MIC value in comparison to tetracycline with MIC value of 31.25 μg/mL. These results suggest that at a lower concentration the crude extract exhibits strong antibacterial activity that is attributable to either a single antibacterial compound present in the crude extract or several compounds working synergistically to elicit a strong antibacterial action (Williamson, 2001; Yarnell, 2015).

#### Cell Membrane Disruption Assay

**FIGURE 4:**
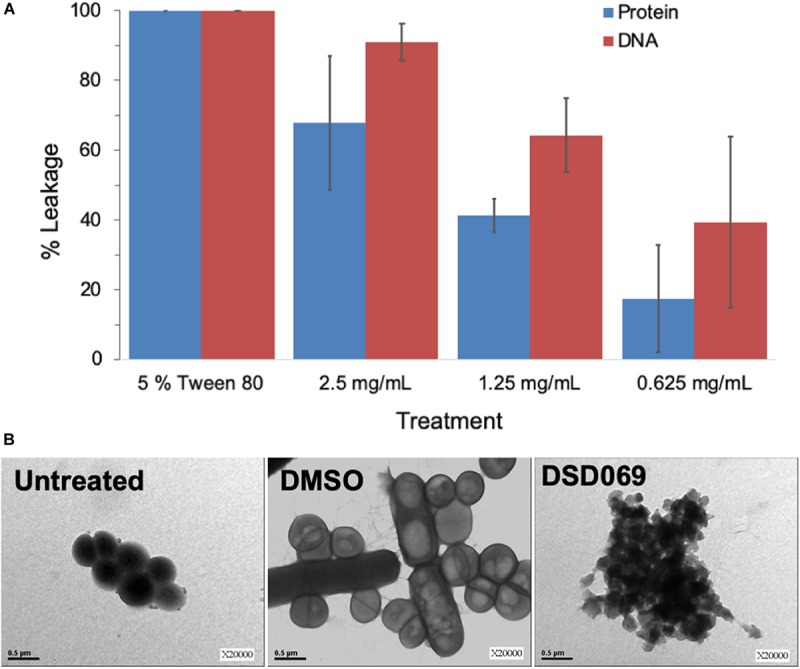
Compromised cell membrane permeability and integrity. Data illustrated are **(A)** percent leakage of the UV_260_ (DNA) and UV_280_ (proteins) absorbing intracellular materials, and **(B)** TEM analysis of *S. aureus* ATCC BAA-44 exposed to different treatments namely, Untreated cells of *S. aureus* ATCC BAA-44 as a control, DMSO as a negative control, and *S. griseorubens* strain DSD069 crude extract (2.5 mg/mL).

Bacterial cell membrane integrity was investigated through a leakage assay by measuring the leaked DNA and proteins. Results showed that the antibacterial activity is associated with membrane damage in a concentration-dependent manner (Figure 4). High absorbance values at 260 and 280 nm suggest leakage of DNA and proteins, respectively, which suggest that bacterial cell membrane integrity may have been compromised, allowing the intracellular materials (DNA and proteins) to leaked out of the cell. The percent of DNA and protein leakage of *S. griseorubens* strain DSD069 crude extract was calculated relative to 5% Tween 80, a known compound that affects cell membrane permeability (positive control) (Brown and Winsley, 1969). The result showed that *S. griseorubens* strain DSD069 crude extract exhibited 85.27% DNA leakage and 67.68% protein leakage at 2.5 mg/mL concentration.

This observation was further validated by TEM analysis. It was found that *S. aureus* ATCC BAA-44 cells exposed to *S. griseorubens* strain DSD069 extract at 2.5 mg/mL for 24 h showed cell membrane disruption (Figure 4B). The cells appeared with an irregular shape, collapsed, translucent, and formed aggregates of shrunken cells. These findings confer the cell membrane damaging effect of *S. griseorubens* strain DSD069 crude extract. While untreated cells and DMSO treated cells appeared to be spherical with smooth cell surface even on dividing cells; hence, indicating that the cell membranes are still intact.

**FIGURE 5:**
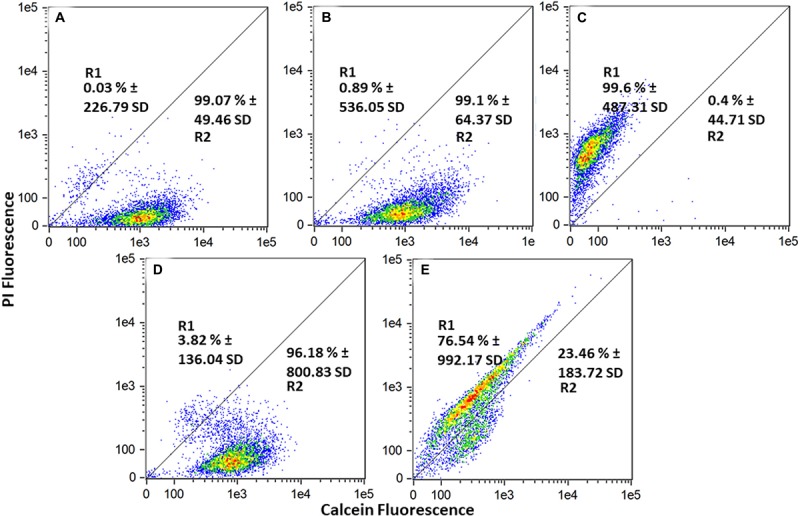
Cell membrane permeability effect of *Streptomyces griseorubens* strain DSD069 crude extract. Data presented are flow cytometer scatter plots profile of various treatments namely: **(A)** Untreated, **(B)** DMSO, **(C)** 70% Ethanol, **(D)** Vancomycin, 7.5 mg/mL, and **(E)** *Streptomyces griseorubens* strain DSD069 crude extract, 2.5 mg/mL. The experiment was done in triplicates.

To further assess the damaging effects of *S. griseorubens* strain DSD069 extract on cell membrane permeability and integrity, flow cytometry experiment using fluorescent dyes, calcein AM, and propidium iodide was performed. Calcein emits green fluorescence when intracellular esterases of live cells convert calcein AM to calcein. In contrast, red-fluorescent propidium iodide can only penetrate bacterial cells with compromised cell membrane permeability or integrity. Cell populations in the flow cytometer dot plots were clustered into two regions, namely dead or membrane-damaged cells (R1), which have strong propidium iodide fluorescence, and live cells (R2), which have strong calcein fluorescence (Wu et al., 2016). The untreated (99.07%) and DMSO-treated (99.10%) cells were mostly live cells, as indicated by high populations of calcein-fluorescing cells (Figure 5). Ethanol-treated cells, on the other hand, exhibited a high cell population (99.6%) fluorescing with propidium iodide, thus indicating that these cells were dead and have damaged cell membranes. After 4 h of incubation, *S. aureus* ATCC BAA-44 cells treated with *S. griseorubens* strain DSD069 crude extract at 2.5 mg/mL (the same concentration used in protein and DNA leakage assay and time kill assay) exhibited 76.54% of dead cells with damaged cell membrane as indicated by propidium iodide fluorescing cells and only 23.46% of calcein fluorescing cells (live cells). On the contrary, vancomycin at 7.5 mg/mL treatment exhibited only 3.82% of the dead and membrane-permeant cell population, and a large population of live cells fluorescing with calcein (96.18%) was observed. Previous reports on vancomycin time-kill kinetics against methicillin resistant *S. aureus* strains suggest onset antibacterial activity after 4 h incubation period (Singh et al., 2009) and more than 90% inhibition after 8 h incubation period (Foster et al., 1986). Moreover, this suggests the slow inhibiting action of vancomycin explaining why only a small percentage (3.82%) of the dead cell population was observed in our experiment.

Collectively, the results of the assays confirm that the loss of viability of multidrug resistant *S. aureus* ATCC BAA-44 when treated with *S. griseorubens* strain DSD069 crude extract is a consequence of cell membrane damage. The destruction of cell membrane is demonstrated by (a) leakage and loss of vital cell constituents, including DNA and proteins, (b) irregular shrinkage of cells, and (c) enhanced membrane permeability as shown by propidium iodide uptake.

### Antibacterial Compounds From *Streptomyces griseorubens* Strain DSD069

To identify the compound/s responsible for the antibiotic activity against the multidrug resistant *S. aureus* ATCC BAA-44, thin layer chromatography (TLC) bioautography was performed. The bioautography assay result suggests that bands with R_f_ values of 0.79 and 0.88 possessed antibacterial activity against *S. aureus* ATCC BAA-44 as indicated by the blue coloration in the bioautogram (Supplementary Figure 3). The bioactive band at R_f_ 0.79 has a characteristic intense orange fluorescence at 365 nm, while the band at R_f_ 0.88 has a dark orange fluorescence suggesting aromaticity.

Purification of the active components was done by passing 88.6 mg of *S. griseorubens* strain DSD069 crude extract through a silica column using dichloromethane followed by methanol-dichloromethane (1:13) as the mobile phase, which yielded 50 fractions (Supplementary Figure 4). The fractions were developed in a silica TLC plate using a methanol-DCM (1:13) solvent system and viewed under UV at 254 and 365 nm. Based on UV activity, fractions 37–42 were pooled together and dried that yielded 12.9 mg. Preparative silica TLC was used to further separate the bioactive bands at R_f_ 0.79 and R_f_ 0.88 and was designated as BAF1 and BAF2, respectively.

**FIGURE 6:**
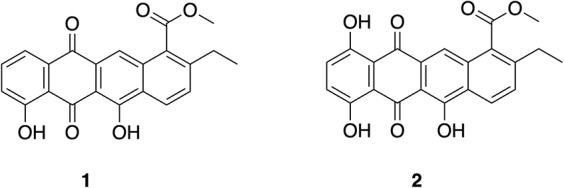
Structures of anthracycline shunt metabolites: Bisanhydroaklavinone **(1)** and 1-Hydroxybisanhydroaklavinone **(2)**.

BAF1 was obtained as a yellow film. The molecular formula was determined as C_22_H_16_O_6_ by high-resolution electrospray ionization mass spectrometry (HRESIMS) (*m/z* 377.10274 [M + H]^+^, calcd. 377.10251), which indicated 15 degrees of unsaturation (Figure 6). MSe and dereplication analysis (Waters UNIFI Scientific Information System^®^ and Chemspider^TM^ database) revealed that BAF1 matched with Bisanhydroaklavinone, 1 (Supplementary Figure 5). On the other hand, BAF2 was also obtained as a yellow film. The molecular formula was determined as C_22_H_16_O_7_ by high resolution electrospray ionization mass spectrometry (HRESIMS) (*m/z* 393.09733 [M + H]^+^, calcd. 393.09743), which indicated 15 degrees of unsaturation (Figure 6). BAF2 matched with 1-Hydroxybisanhydroaklavinone, **2** (Supplementary Figure 6).

To investigate its antibacterial activity, microbroth susceptibility assay was performed at 100 mg/mL concentration against *S. aureus* ATCC BAA-44. Our results showed that **1** and **2** exhibited antibacterial activity of 92.09% and 99.14%, respectively. In comparison, tetracycline showed 93.40% inhibition at 50 μg/mL (Supplementary Figure 7).

The antibacterial activity against *S. aureus* ATCC BAA-44 was further confirmed by microbroth dilution assay to determined its minimum inhibitory concentration (MIC). Compound **1** strongly inhibits the growth of *S. aureus* ATCC BAA-44 as shown by its minimum inhibitory concentration (MIC) value of 6.25 μg/mL. Compound **2** and tetracycline displayed weak antibacterial activity against *S. aureus* ATCC BAA-44 with MIC value of 50 μg/mL and 50 μg/mL, respectively (Supplementary Figure 8).

### Anthracycline Shunt Metabolites: Bisanhydroaklavinone (**1**) and 1-Hydroxybisanhydroaklavinone (**2**)

Compounds **1** and **2** are shunt metabolites of *Streptomyces* anthracycline glycosides such as the aclacinomycins (e.g., aclarubicin) (Eckardt and Bradler, 1965; Oki et al., 1975; Hori et al., 1977; Niemi et al., 1999), rhodomycins (e.g., daunorubicin and doxorubicin) (Brockmann and Franck, 1955; Arcamone et al., 1969; Grein, 1987), and cinerubins (cinerubin A and cinerubin B) (Corbaz et al., 1957; Eckardt et al., 1989). These anthracycline glycosides, however, are well known anticancer drugs (Egorin et al., 1981; Nitiss et al., 1997). Most of these bioactive anthracyclines are glycosides, containing one or more sugar moieties attached to the aglycone, which was reported to be an important structural requirement for their anticancer activities (Clark et al., 2004).

Compound **1** was previously reported to be a biosynthetic shunt product of block mutant strains of *Streptomyces galilaeus* as part of a study to understand the biosynthesis of aclacinomycins (Oki et al., 1980; Tobe et al., 1982). On the other note, **2** was reported as an isomer of Bisanhydro-δ-rhodomycinone and Bisanhydro-ε-rhodomycinone (Brockmann and Brockmann, 1963). Compound **2** is also known as η-pyrromycinone, a non-glycosidic compound isolated from a soil-derived *S. galilaeus* from Isle of Hidensee, Germany together with anticancer cinerubin A (Schlegel et al., 1987). No prior studies on the anticancer and antibiotic activity of **1** and **2** has been reported.

Chemical synthesis revealed that **1** could be derived from aklavinone and **2** from ε-pyrromycinone through hydrobromide/glacial acetic acid reaction and from 7-desoxyaklavinone and ζ-pyrromycinone through Pd/Mohr or Pd/Kohle reaction, respectively (Eckardt, 1967). This chemical synthesis work supports the report that **1** and **2** can be double dehydration products of anthracyclinones produced by *Streptomyces* strains (Brockmann et al., 1965). This dehydration process, particularly on ring D of tetracenedione chromophore of these major aglycones, readily takes place in acidic conditions that resulted in the formation of 7,9-diols, which subsequently undergo bis-hydration further resulting to the aromatization of ring D (Lown, 1993). Compound **2** is hydroxylated at C-1 position of tetracenedione nucleus. This hydroxylation event was in the biosynthesis of anticancer cinerubins where AclR enzyme catalyzes the hydroxylation at C-1 position of aklavinone. The mechanism and reaction pathway of C-1 modification of the aglycone is not fully understood (Beinker et al., 2006). Interestingly, studies on the biosynthetic gene cluster of a similar anthracycline anticancer derivative, nogalomycin, involved a two-component monooxygenase SnoaW/SnoaL2 enzyme that is responsible for the hydroxylation at C-1 tetracenedione nucleus of aklavinone to 1-hydroxyaklavinone prior to glycosylation to form nogalomycin (Bechthold and Yan, 2012).

**SCHEME 1:**
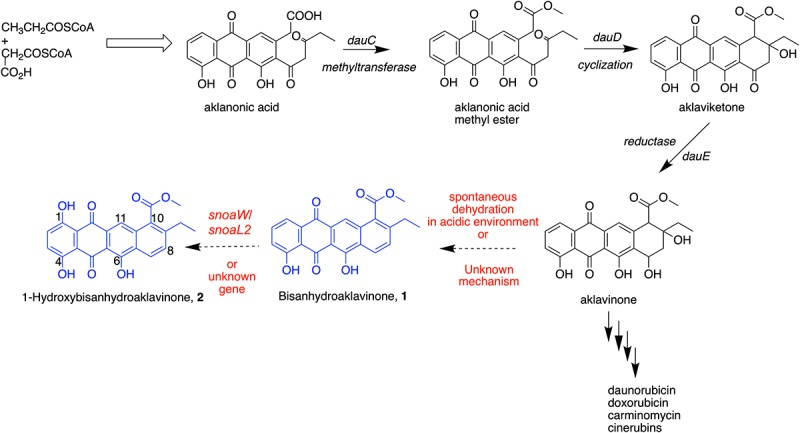
Putative biosynthetic pathway of 1 and 2 in marine sediment-derived *Streptomyces griseorubens* strain DSD069.

In this study, we found that **1** and **2** are metabolites accumulated in the biomass of marine sediment-derived *S. griseorubens* strain DSD069. Based on this finding, we propose in Scheme 1 that the accumulation of dehydrated, non-glycosylated anthracyclinone compounds **1** and **2** was influenced by the acidity of the growth medium used in this study (pH 5.0–6.0) or perhaps other unknown mechanisms that favors the spontaneous dehydration and subsequent aromatization of ring D in the tetracenedione chromophore. This proposed mechanism is consistent with the previous report on spontaneous dehydration of aklavinone in an acidic environment to form Bisanhydroaklavinone (**1**) (Lown, 1993). Moreover, perhaps SnoaW/SnoaL2, AclR enzyme (C-1 hydroxylase) or similar functioning enzyme may have been well expressed in marine sediment-derived *S. griseorubens* strain DSD069 that may have favored the formation of 1-Hydroxybisanhydroaklavinone (**2**).

## Conclusion

The findings in this study provide evidence on the potential of marine sediment-derived *Streptomyces* strains from Romblon, Philippines as a source of antibiotics against *S. aureus* ATCC BAA-44. Here, we found that *S. griseorubens* strain DSD069 exhibited its highest antibacterial activity by destroying the cell membrane of *S. aureus* ATCC BAA-44 as demonstrated by protein and DNA leakage and loss of vital cell constituents, irregular shrinkage of cells, and increase membrane permeability. We found that anthracycline shunt metabolites **1** and **2** are the major compounds produced by *S. griseorubens* strain DSD069 that are responsible for its antibacterial activity and plausibly causing the membrane damaging activity. The identification of the molecular target of anthracycline shunt metabolites **1** and **2** as well as their toxicity profiles warrants further investigation. Moreover, we have demonstrated that Philippine marine sediment-derived *S. griseorubens* strain DSD069 is a natural bacterial strain that can produce anthracycline shunt metabolites **1** and **2,** with potential as antibiotic leads to combat ABR. These anthracycline shunt metabolites were accumulated during the fermentation process. We proposed that the intermediate aglycone aklavinone in the biosynthesis of anticancer anthracycline derivatives namely doxorubicin, daunorubicin, and cinerubins underwent spontaneous double dehydration to afford **1**, which subsequently converted to **2** via C-1 hydroxylation. These biosynthetic events are driven by mechanisms that are not yet fully understood in this study. Thus, the intriguing biosynthetic machinery found in Philippine marine-sediment derived *S. griseorubens* strain DSD069 that produce these shunt metabolites are interesting areas to look into for future studies.

## Data Availability Statement

The datasets generated for this study can be found in the GenBank with accession number MN818600.

## Author Contributions

DD conceptualized the study. DD and JS designed the experiments. MP, AS, ES, and ZL performed the experiments. MP, AS, ES, ZL, JS, and DD analyzed the data. MP, JS, and DD wrote and edited the manuscript.

## Conflict of Interest

ZL was employed by Waters Pacific Pte Ltd.

The remaining authors declare that the research was conducted in the absence of any commercial or financial relationships that could be construed as a potential conflict of interest.
