# Supplementary material for: Anthracycline Shunt Metabolites From Philippine Marine Sediment-Derived Streptomyces Destroy Cell Membrane Integrity of Multidrug-Resistant Staphylococcus aureus
Source: Front Microbiol. 2020 Apr 24;11:743. doi: 10.3389/fmicb.2020.00743 (PMC7193051; doi:10.3389/fmicb.2020.00743)
Supplement: Supplementary file 1 [file Data_Sheet_1.docx]

**Supporting Information**

**SI Table 1. Antibacterial Activities of *Streptomyces* *griseorubens* strain DSD069 .**

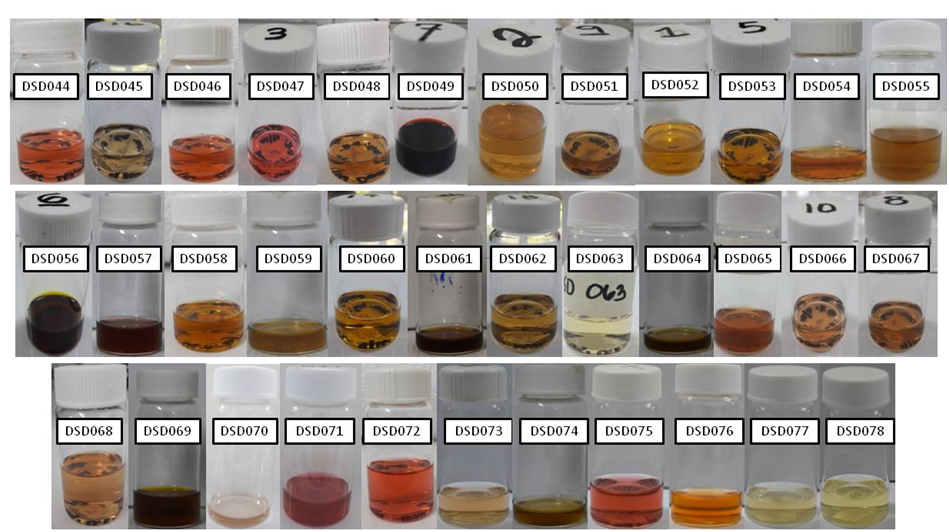


**SI Figure 1. Marine Sediment-derived Actinobacterial Crude Extracts.** Biomass of pure Actinobacterial isolates were extracted with EtOAc and subsequently partitioned in water. The crude extracts were concentrated and dried *in vacuo*.

**
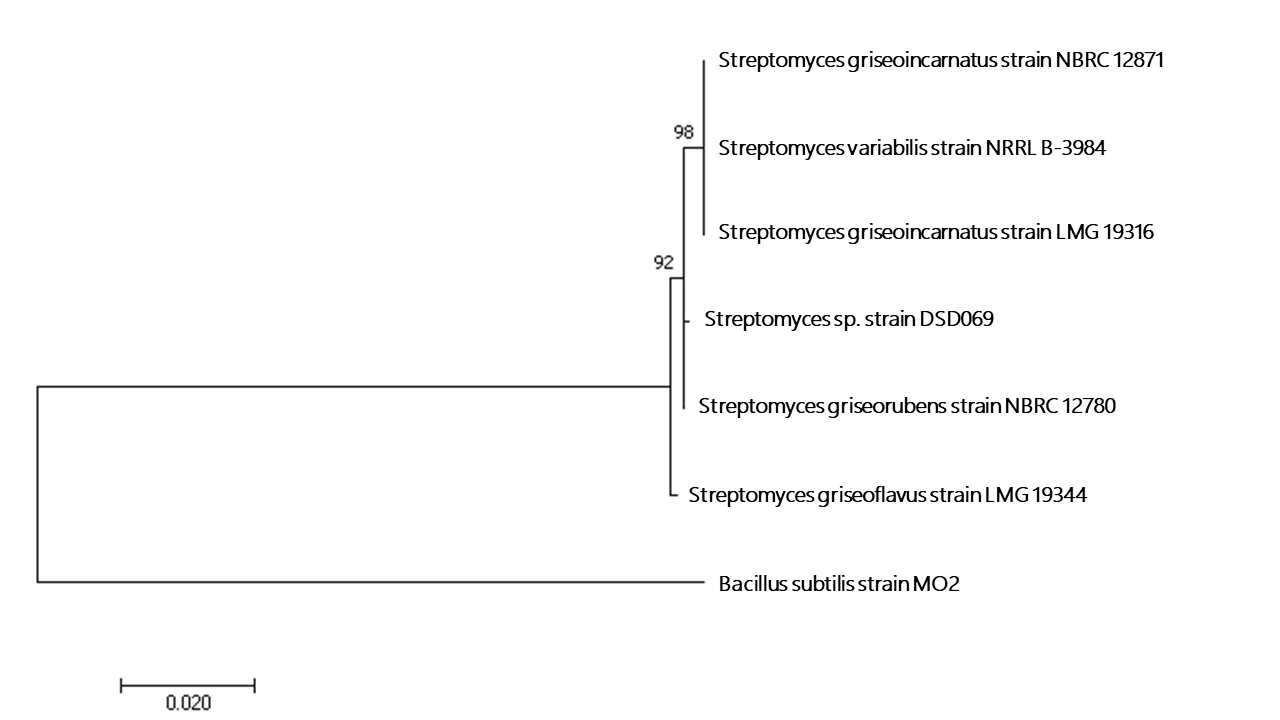
**

**SI Figure 2. Molecular phylogenetic analysis of *Streptomyces* *griseorubens* strain DSD069 by maximum likelihood method.** Data presented are the percentage of trees in which the associated taxa clustered together is shown next to the branches. FASTA format of the gene sequence of the closely related strain based on the result of BLAST was subjected to analysis using MEGA version 7 software using bootstrap value of 1000.


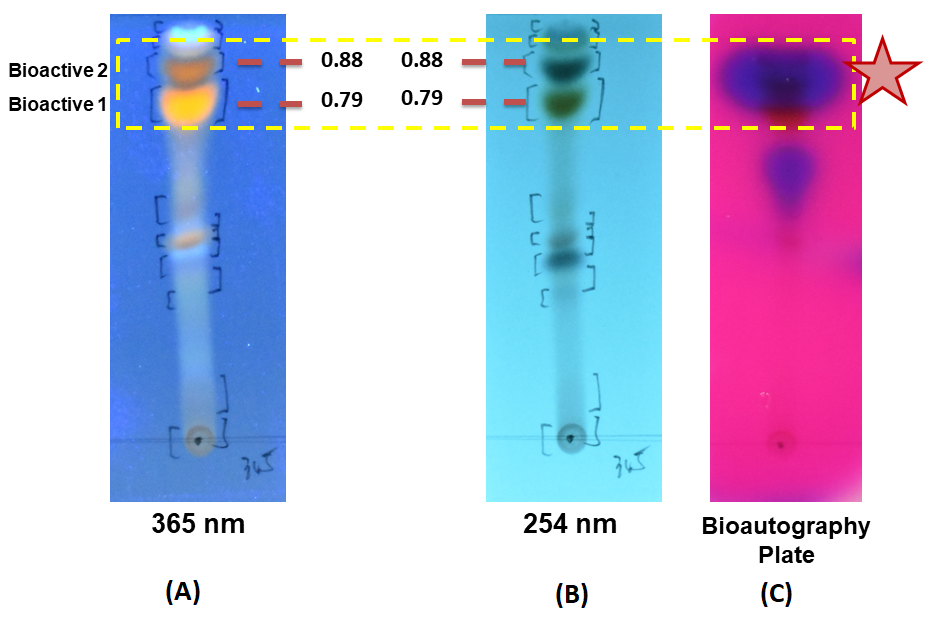


**SI Figure 3. Bioautography result of *Streptomyces griseorubens* strain DSD069 against multidrug-resistant *S. aureus ATCC BAA-44***. (**A**) TLC plate viewed under UV 365 nm, (**B**) TLC plate viewed under UV 254 nm, and (**C**) Bioautography plate. TLC chromatogram of *Streptomyces griseorubens* strain DSD069 crude extract was overlaid with soft agar seeded with *S. aureus* ATCC BAA-44. The bioautogram plate was incubated for 24 h at 37 ^0^C. After incubation, the bioautogram plate was flooded with 0.015% resazurin in PBS, and the reaction was allowed to occur for 1 h. Blue spots on the bioautography plate marked with a star indicate presence of dead cells.


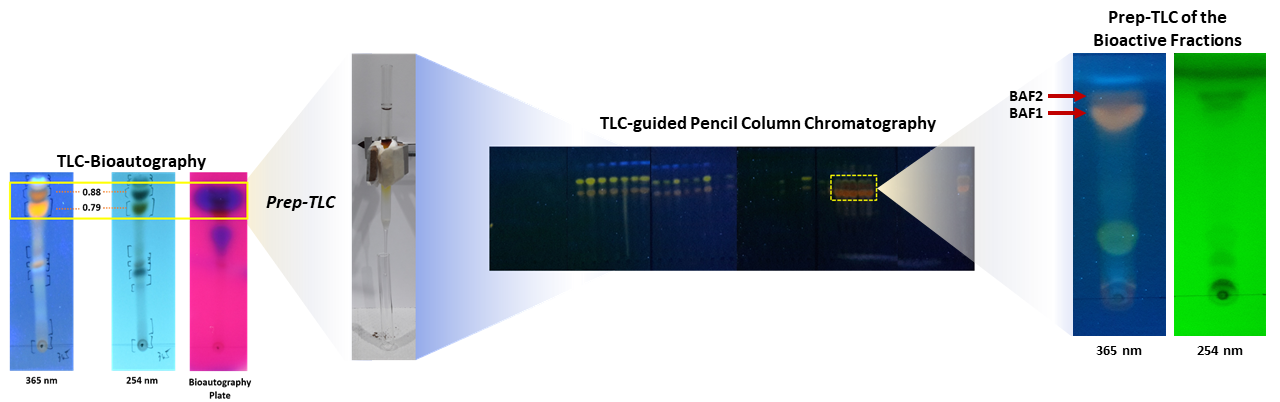


**SI Figure 4. TLC-Guided Purification of the bioactive fractions of *Streptomyces griseorubens* strain DSD069.** Diagram presented illustrate the flow of how the bioactive fractions was obtained.

**SI Figure 5. HRMS and MSe spectra and analysis of Bisanhydroaklavinone, 1**

**SI Figure 6. HRMS and MSe spectra and analysis of 1-Hydroxybisanhydroaklavinone, 2**

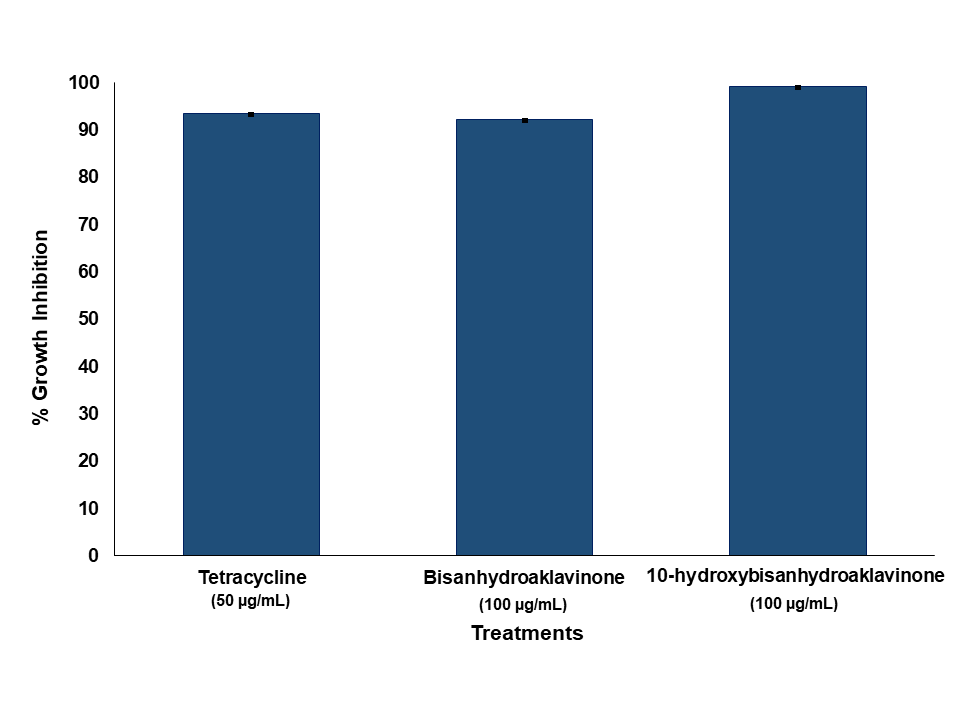


**SI Figure 7. Percent Growth Inhibition of the bioactive fractions against multidrug-resistant *Staphylococcus aureus* ATCC BAA-44.** Data illustrates the percent growth inhibition, against *S. aureus* ATCC BAA-44 of bisanhydroaklavinone and 1-hydroxybisanhydroaklavinone. The experiment was done in triplicates and performed in three trials.


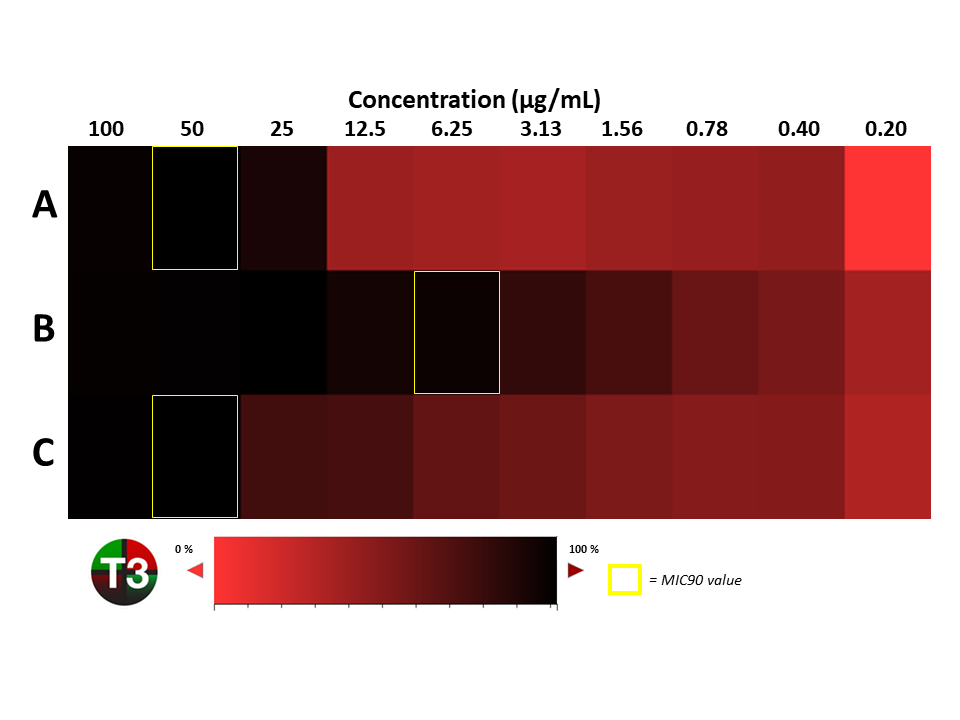


**SI Figure 8. Heat Map of the Minimum inhibitory concentration of the bioactive fractions.** Data presented are MIC_90_ (solid yellow outline) of the different treatments. Multidrug-resistant *S. aureus* ATCC BAA-44 (1 x 10^6^ cfu/mL) was exposed with serially diluted concentrations of (**A**) tetracycline, (**B**) Bisanhydroaklavinone, and (**C**) 1-Hydroxybisanhydroaklavinone for 18-24 h at 37 ^0^C. Bacterial densities were measured spectrophotometrically after incubation period and corresponding % growth inhibition was calculated (Babicki, et al., 2016 & Haarman, et al., 2015). N=3 trials in triplicates.
